# Non-mammalian models in behavioral neuroscience: consequences for biological psychiatry

**DOI:** 10.3389/fnbeh.2015.00233

**Published:** 2015-09-08

**Authors:** Caio Maximino, Rhayra Xavier do Carmo Silva, Suéllen de Nazaré Santos da Silva, Laís do Socorro dos Santos Rodrigues, Hellen Barbosa, Tayana Silva de Carvalho, Luana Ketlen dos Reis Leão, Monica Gomes Lima, Karen Renata Matos Oliveira, Anderson Manoel Herculano

**Affiliations:** ^1^Laboratório de Neurociências e Comportamento, Departamento de Morfologia e Ciências Fisiológicas, Campus VIII – Marabá, Universidade do Estado do ParáMarabá, Brazil; ^2^Universität Duisburg-EssenEssen, Germany; ^3^Laboratório de Neurofarmacologia Experimental, Instituto de Ciências Biológicas, Universidade Federal do ParáBelém, Brazil

**Keywords:** behavioral models, anamniotes, invertebrates, biological psychiatry, teleost fish, sauropsida

## Abstract

Current models in biological psychiatry focus on a handful of model species, and the majority of work relies on data generated in rodents. However, in the same sense that a comparative approach to neuroanatomy allows for the identification of patterns of brain organization, the inclusion of other species and an adoption of comparative viewpoints in behavioral neuroscience could also lead to increases in knowledge relevant to biological psychiatry. Specifically, this approach could help to identify conserved features of brain structure and behavior, as well as to understand how variation in gene expression or developmental trajectories relates to variation in brain and behavior pertinent to psychiatric disorders. To achieve this goal, the current focus on mammalian species must be expanded to include other species, including non-mammalian taxa. In this article, we review behavioral neuroscientific experiments in non-mammalian species, including traditional “model organisms” (zebrafish and *Drosophila*) as well as in other species which can be used as “reference.” The application of these domains in biological psychiatry and their translational relevance is considered.

## Introduction

The use of model organisms is paramount in the behavioral neurosciences and its ramifications into biological psychiatry. Among these organisms, rats, and mice are still the most widely used, although other fields of the neurosciences use different species. This almost exclusive focus on rodents is problematic from the epistemic point of view, and behavioral neuroscience could profit from the inclusion of more species in its analysis. In fact, since the comparative aspect is a strong argument in favor of using non-human animals in behavioral research, the expansion of species is important to strengthen that argument. Moreover, insights gained from other species—including model organisms and “reference species” (Striedter et al., 2014)—can help understand what is generalizable and what is species-specific. In this Review, we highlight the role of model organisms and reference species in the behavioral neurosciences and discuss some advances, advantages and disadvantages of using a few choice species in behavioral research with consequences for biological psychiatry.

## Model organisms in behavioral neuroscience

“the fish is a frog…is a chicken…is a mouse”

(Kimmel, 1989)

### Foundational issues

Before the discussion about model organisms advances, some definitions must be settled. In the life sciences, the term “model organism” refers to a species that is used in an attempt to understand particular biological phenomena (Fields and Johnston, 2005). From an epistemic point of view, a model organism acts as a stand-in for other organisms:

[…] model organisms are always taken to represent a larger group of organisms beyond themselves, and hence rely on very particular types of claims about their (potential) representational scope. […] The actual relationships between the model organism and this larger group often are very ill-articulated in the earliest stages of model organism work, and do not necessarily hinge on particular claims about genetic conservation or precise knowledge of the phylogenetic placement of a particular organisms in relationship to others (Ankeny and Leonelli, 2011, p. 318).

Another important epistemic characteristic of model organisms is that, differently from what Ankeny and Leonelli (Ankeny and Leonelli, 2011; Leonelli and Ankeny, 2013) call “experimental organisms,” they target a wide range of systems and processes occurring in living organisms, including genetics, development, physiology, behavior, evolution, and ecology. In this sense, model organisms are “material analogies” (Hesse, 1963) which, although not faithfully mirroring their target, represent other organisms at the most basic levels (Ankeny and Leonelli, 2011; Leonelli and Ankeny, 2013).

The concept of model organism is not specific to any field of the life sciences; the concept of “animal model,” in contrast, is more common in the behavioral sciences. This term refers not to an organism, but to the conjunction of apparatuses and manipulations used to represent a given behavioral (dys)function in a different species than the target (van der Staay, 2006; Nelson, 2012). Animal models are most widely used in the fields of behavior genetics, biological psychiatry, experimental psychopathology, and neuropsychopharmacology, where they are used to generate biological (physiological or genetic) hypotheses regarding psychiatric disorders, to investigate the psychological aspects of the disorder, or to screen for potential psychiatric drugs (McKinney and Bunney, 1969; Willner, 1991; Wright, 2002).

LaFollette and Shanks (1995) defined two categories of animal models: causal analog models, with which experimenters test causal mechanisms in a model and then extrapolate, by analogy, to the human condition; and hypothetical analogical models, which have the function of generating novel hypotheses. LaFollette and Shanks (1994, 1995) argued that the assumption of interchangeability between non-human animals and humans is weak at best, and therefore animal models are better used to generate novel hypotheses. However, it has been argued that models with predictive validity and construct validity show biological and translational relevance, and therefore can be used as causal analog models (van der Staay, 2006).

While the majority of animal models use model organisms as subjects—especially rats and mice (Griebel and Holmes, 2013) –, that is not a strict requirement to model a given psychiatric disorder. In fact, the definition of an animal model as an experimental preparation is neutral with regard to the extension of what is being modeled and to which species is targeted (Wright, 2002). Nonetheless, most assumptions of model organism research also inform animal modeling (Kalueff et al., 2008; Maximino et al., 2010c; de Mooij-van Malsen et al., 2011; Kas et al., 2011; Stewart and Kalueff, 2014). While the aspects of pharmacological isomorphism, ethological consistency, and symptomatology are central to reasoning with animal models (Willner, 1991; van der Staay, 2006; Kalueff et al., 2007; Belzung and Lemoine, 2011), “[t]he arguments for evolutionary relationships, genetic homologies, and physiological similarities also are part of the epistemic infrastructure that supports the use of animal models” (Nelson, 2012, p. 16). In particular, some authors (Blanchard and Blanchard, 1988; Maximino et al., 2010c; de Mooij-van Malsen et al., 2011; Kas et al., 2011; Stewart and Kalueff, 2014) advocate the use of species-specific behavioral and physiological phenotypes as endpoints for assessing the effects of manipulations across multiple species. This, of course, necessitates the model to be embedded in a theoretical framework which will guide the choice of endpoints to be analyzed and validated (McNaughton and Zangrossi, 2008; Maximino et al., 2010c).

### Expanding the breadth of species

The choice of species is usually guided by practical advantages—throughput, fertility, developmental speed, availability of genomic and transcriptomic data—and, to a great extent, to the existence of well-established research communities and data availability (Fields and Johnston, 2005; Ankeny and Leonelli, 2011; Leonelli and Ankeny, 2012). In the neurosciences, further criteria are the amenability to genetic manipulations and relative simplicity of the nervous system. These advantages are more extensive in molecular neurosciences, including neurogenetics and developmental neuroscience, and historically gave rise to a handful of model organisms—*viz*, humans, macaques, rats, mice, zebrafish, *Xenopus, Drosophila* and *C. elegans*. Extensive databases of gene expression for flies [http://flybase.org/], frogs [http://www.xenbase.org], humans [http://human.brain-map.org/], mice [http://mouse.brain-map.org/], worms [http://www.wormbase.org/], and zebrafish [http://zfin.org/] are already available, allowing for the comparison of basal expression levels in different brain areas. These data can be used in comparative neuroanatomy to refine homology propositions (Engert, 2014; Mitra, 2014; Striedter et al., 2014), which is essential for circuit approaches in behavioral neuroscience. These data can also be used to mine for the neuroanatomical localization of psychiatric disorder-related genes in different model organisms (de Mooij-van Malsen et al., 2011; Kas et al., 2011). These neuroinformatic approaches exemplify the power of current data tools available for well-established model organisms in the neurosciences.

In addition to studying well-established model species, behavioral neuroscience could benefit from focusing on other, carefully chosen species to amplify the field of discovery. Striedter et al. (2014) used the term “reference species” to define “carefully selected species from phylogenetically widely spaced vertebrate and invertebrate groups” for comparative neuroanatomy. These species would then serve two purposes: as substrates for broad comparisons across all animals to identify nervous system fundamentals and as anchors for more fine-grained analyses within their particular taxon to assess the meaning of variation in whole brains and functional subsystems (Striedter et al., 2014, p. 5).

**Figure 1:**
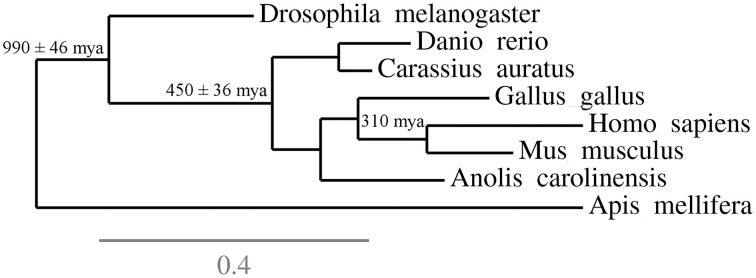
**Phylogenetic context of some “reference species” that can be used in behavioral neurosciences**. The figure underlines the position and phylogenetic distances of a few species in relation to each other, and suggest how this information can be used to inform the selection of organisms for research. For example, while most research in the behavioral neurosciences is performed using rodents, selecting a species from an outgroup—for example, chicks or lizards—could inform researchers on evolutionary conservation of biobehavioral traits in mammals. These informations complement the usual criteria for species choice (ease of reproduction, rapid generation time, etc.) and the availability of behavioral and physiological assays.

While the authors' focus was comparative and evolutionary neuroanatomy, their conclusions and recommendations can be extended to behavioral neuroscience insofar as both areas can profit from comparing taxa to infer how variations in one domain (gene expression, connectivity, activation patterns) relates to variation in behavior (Engert, 2014; Mitra, 2014; Striedter et al., 2014). Striedter et al. (2014) suggest that well-established model organisms be included among these species due to the availability of resources for their study, but point that other reference species should also be selected based on a few criteria. Thus, reference species are not “models for some other species, but […] a basis for comparisons that may reveal both similarities and differences” (Striedter et al., 2014, p. 5) The criteria for choosing a reference species are not established *a priori*, but might include phylogenetic position (Figure 1) and accumulation of significant data and methodological developments (Hale, 2014; Striedter et al., 2014); the ultimate goal is to allow the emergence of a comprehensive understanding of specific behavioral functions in different species and its specific relationships to brain structure and activity (Striedter et al., 2014; Hale, 2014).

The assumption of conservation is also best studied in a comparative framework. While this assumption is essential for projecting even rodent data toward humans, it has not been tested for most behavioral domains which are relevant for biological psychiatry (Panksepp et al., 2002; Pollen and Hofmann, 2008). It has been argued that, at least in anxiety research, construct validity is dependent on the assumption of evolutionary conservation (Maximino et al., 2010c); thus, to increase construct validity in animal models, testing the predictions of this assumption is highly desirable (McNaughton and Zangrossi, 2008). In this sense, while a common practice in behavioral neuroscience, using data from a single species (no matter how basal) to infer the ancestral state is unfeasible (Garland, 2001). As we will see along this article, while currently the most basal vertebrate used in biological psychiatry is zebrafish, a comparison with closely-related species (e.g., goldfish or guppy) as well as more basal vertebrates (e.g., sharks or lampreys) is necessary to establish the ancestral state of a given neurobehavioral trait in vertebrates. Studying species phylogenetically located near the origin of vertebrates, such as acorn worms and amphioxus, could also be useful “for comparisons that span both vertebrate and invertebrate nervous systems” (Striedter et al., 2014). As a result, behavioral neuroscience could clearly profit from the adoption of other, non-model, “reference” species in the same way that neuroanatomy had.

## Behavioral research in non-mammalian species: Relevance to biological psychiatry

Important as it may be for comparative and evolutionary neuroscience, cross-species research in the behavioral neuroscience is still incipient. Some approaches have been proposed which include comparative research in order to clarify genotype-phenotype relationships, thereby increasing the translational value of animal behavior in relation to human neuropsychiatric disorders (Kalueff et al., 2008; LaPorte et al., 2008; de Mooij-van Malsen et al., 2011; Kas et al., 2011). The main rationale is based on the utility of model organisms in each specific domain. For example, rodent stress responses are driven by corticosterone, while zebrafish and humans use cortisol (Steenbergen et al., 2011). In addition to this critical but utilitarian aspect, the expansion toward “reference species”—especially non-mammalian species—could allow for uncovering conserved and divergent mechanisms underlying pathogenesis (Kalueff and Stewart, 2014; Stewart and Kalueff, 2014). An example of conservation at the genomic and functional levels is the regulation of neurosteroidogenesis in relation to the anxiolytic effect of fluoxetine in both zebrafish (Wong et al., 2013) and mice (Pinna, 2010); an example of divergence is the serotonergic system, which show both duplicated genes and nuclei other than the raphe a multiplication of nuclei in fish in relation to rodents but is equally involved in anxiety and fear (Lillesaar, 2011; Herculano and Maximino, 2014). In both cases, a derivative endophenotype (Kalueff and Stewart, 2014) results from domain interaction that is conserved or divergent.

In what follows, we will exemplify conserved (or at least convergent) phenotypes that can be found in non-mammalian model organisms and in proposed reference species, as well divergent phenotypes which are found in model and reference species which could be useful for neuroscientific research. While not attempting to be a comprehensive review of every possible use of non-mammalian species in biological psychiatry, some specific examples were chosen based on phylogenetic position, data availability, and ease of use in laboratory settings. In most of the cases, pharmacological correlates are the strongest argument for the use of that species and behavioral model, which underlines the need to reinforce construct validity (the theoretical network behind the model) and face validity (the neurobehavioral isomorphism between the model and the target pathology).

### Teleost fish behavior

#### Anxiety-like behavior in cyprinids

Human psychiatric disorders associated with anxiety, stress and phobic states result from abnormalities in neurobiological processes, inducing characteristic behavioral responses (Panksepp, 2004, 2006; LeDoux, 2012). Recently, diverse behavioral tests for anxiety, fear, and stress were proposed using teleost fish, among which zebrafish and goldfish stand out (Maximino et al., 2010d; Stewart et al., 2011a; Steenbergen et al., 2011). The potential of these species as model organisms for the analysis of genetic and biological mechanisms of fear and anxiety in vertebrates is beginning to be realized (Kalueff et al., 2014a; Stewart et al., 2014b). Moreover, other fish species—including goldfish and guppies—are being used in behavioral neuroscience, expanding the comparative breadth of teleost fish in this field (Hall et al., 2014).

Zebrafish is a small cyprinid that has long been used as a model organism in developmental biology and genetics (Signore et al., 2009). Its physiology is comparatively simple, making the species adequate for high-throughput investigation in pharmacology, toxicology, behavioral genetics and pharmacogenomics (Kokel and Peterson, 2008; Gerlai, 2010, 2015; Kalueff et al., 2014b). Zebrafish also presents neuroanatomical landmarks and neurotransmitter systems which are very similar to those observed in mammals (Rinkwitz et al., 2011; Kalueff et al., 2014b).

Due to the many inherent advantages of zebrafish as a model organism—including low cost, easy manipulation and upkeep in relation to other vertebrate models, and 70–80% genetic homology with humans (Table 1)—zebrafish are increasingly useful in fields such as behavior genetics (Gerlai, 2003; Norton and Bally-Cuif, 2010). Although the degree of genetic homology with humans is not as high as in rodents, it is favorable in comparison with other genetically tractable organisms such as *Drosophila melanogaster* and *Caernohabditis elegans* (Kokel and Peterson, 2008). Despite the rising popularity of zebrafish in biological psychiatry (Griebel and Holmes, 2013; Stewart et al., 2014a), behavioral analyses still lack a more torough study (Gerlai, 2003, 2010, 2014, 2015).

**Table 1:** **Advantages and disadvantages of zebrafish and goldfish as models in behavioral neuroscience**.

***Danio rerio (Zebrafish)***	***Carassius auratus (Goldfish)***
**GENERAL ADVANTAGES**	
•Model organism in developmental biology •Rapid generation time •Cost effective/high density stocking •External development •Behaving larvae at 4–7 dpf	•Rapid generation time •Closely related to zebrafish •Cost effective/high density stocking
**IMAGING/NEUROANATOMY**	
•Small size ideal for microscopy (esp. larvae) •Non-invasive brain observation and manipulation due to transparency (larvae and *casper* mutants) •Conservation of major nuclei/brain regions: arcuate nucleus, preoptic area, hippocampus, amygdala, raphe, etc. •Compact neuronal network revealable by two-photon or confocal imaging •Live imaging with genetically encoded calcium indicators •GAL4/UAS enhancer trapping for neuroanatomical determination and pharmacogenetic ablation •Small adult brain size allows reduced number of sections for histological analysis	•Larger size suitable for ablation techniques and *in vivo* electrophysiology •Conservation of major nuclei/brain regions comparable to that of zebrafish •Medium adult brain size still allows for reduced number of sections for histological analysis
**BEHAVIOR**	
•Well-established assays for anxiety/fear/stress, learning, impulse control •Larval assays for high-throughput screening	•Well-established assays for learning and aversive control
**GENOMIC/GENETIC RESOURCES**	
•More than 1.5 million sequenced genes •More than 75,000 annotated gene expression patterns, including miRNAs •Transgenesis using retroviral and transposon vectors •Rapid mutagenesis (TILLING, ENU screens, insertional mutagenesis, zing finger nucleases, TALENs, CRISPR/Cas) •Large collection of mutants •Rapid gene knockdown using antisense morpholinos	
**PHARMACOLOGY/PHYSIOLOGY**	
•Conservation of pharmacological targets •Conservation of classic neurotransmitters (monoamines, amino acids) •Conservation of most neuropeptides (e.g., ACTH, CRF) •Conservation of immediate early genes (e.g., *cfos, jun, homer*)	•Conservation of classic neurotransmitters (monoamines, amino acids) •Conservation of most neuropeptides (e.g., CRF, PACAP, VIP) •Physiological techniques for awake, behaving animals (e.g., ECGs) •Field potential EEGs available
**DATABASES**	
•ZFIN: http://zfin.org •Zebrafish Atlas: http://zfatlas.psu.edu •Zebrafish Brain Atlas: http://www.zebrafishbrain.org •Virtual Brain Explorer for Zebrafish (ViBE-Z): http://vibez.informatik.uni-freiburg.de •Zebrafish Neurophenome Database (ZND):	
**DISADVANTAGES**	
•No inbred strains •Small size for tissue samples and microdialysis •Some anatomical homologies lacking (e.g., nucleus accumbens) or in dispute (e.g., cortex) •Some neuropeptides not conserved (e.g., NPS) •No homologous recombination •Some early genes not found (e.g., *arc*) •Classical electrophysiological tools not well-developed; no functional MRI •Some duplicated genes (e.g., 5-HT_1AA_ and 5-HT_1AB_) •No physiological techniques for awake, behaving animals	•No inbred strains •Lack of established genetic/genomic tools •Small size for microdialysis •Evolutionary distance from humans •Some anatomical homologies lacking (e.g., nucleus accumbens) or in dispute (e.g., cortex) •Retention of duplicated genes unknown

Experiments using zebrafish larvae have been widely used, given that complex behavior appears in this species from 4 to 5 days post fertilization (dpf); these behaviors include prey capture, avoidance, phototaxis, and thigmotaxis, which are readily quantifiable in automated setups (Ahmad et al., 2012). Many studies suggest that avoidance and thigmotaxis can be used as measures of anxiety in zebrafish, and stimulus control of avoidance and thigmotaxis in zebrafish larvae is similar to that of anxiety in humans (Richendrfer et al., 2012).

Zebrafish possess all the “classic” neurotransmitters found in vertebrates (Rinkwitz et al., 2011), and its neuroendocrine system allows for different physiological stress responses (Steenbergen et al., 2011; Pavlidis et al., 2015). In adult animals, two important methods—the novel tank test (Cachat et al., 2010) and the light/dark test (Maximino et al., 2010b)—have been proposed to analyze anxiety-like behavior. In the first case, the animal is introduced to a novel environment, typically adjusting its spatial distribution in a “diving” response that tends to habituate with decreasing novelty and is accompanied by freezing and erratic swimming (Egan et al., 2009; Cachat et al., 2010, 2011; Wong et al., 2010). In the light/dark test, the apparatus is composed of a preferred black compartment and a non-preferred white compartment, and the preference for the black compartment (which does not habituate; Maximino et al., 2010a) is accompanied by risk assessment and, when the animal enters the white compartment, erratic swimming, thigmotaxis and freezing (Maximino et al., 2014d). Both tests show considerable pharmacological isomorphism (Cachat et al., 2011; Stewart et al., 2011b,c; Maximino et al., 2014d) and have been successfully adapted to other species, including goldfish (Maximino et al., 2007, 2010b; Kang et al., 2011c; Nakamachi et al., 2014).

The GABAergic system, a key regulatory element in experimental and clinical anxiety, is well-documented in zebrafish; its inhibition produces anxiogenic-like effects in zebrafish (López-Patiño et al., 2008; Ellis et al., 2012), and positively modulating GABAergic signaling with benzodiazepines or pentobarbital leads to sedation (Kokel et al., 2010; Stewart et al., 2011c; Gupta et al., 2014) and/or anxiolysis (Bencan et al., 2009; Maximino et al., 2011b; Stewart et al., 2011b,c; Vada et al., 2015). In goldfish, a similar pattern is also observed (Matsuda et al., 2011b; Nakamachi et al., 2014).

Serotonin (5-HT) mechanisms were strongly implicated in anxiety in humans and non-human animals (Maximino, 2012). Although the serotonergic system is not anatomically or genetically conserved (Lillesaar, 2011; Maximino et al., 2013a; Herculano and Maximino, 2014), there is some evidence for functional conservation. Extracellular 5-HT levels are positively correlated with anxiety-like behavior in the light/dark test and negatively correlated in the novel tank test (Maximino et al., 2013c). Moreover, the anxiolytic-like effect of drugs targeting different systems is associated with their ability to decrease serotonin turnover in the zebrafish brain (Maximino et al., 2014d). Drugs which increase serotonin levels increase anxiety-like behavior in the light/dark test (Maximino et al., 2013c; Herculano and Maximino, 2014) and decrease it in the novel tank test (Maximino et al., 2013c; Stewart et al., 2013). In the light/dark test, exposure to an aversive olfactory stimulus (alarm substance) greatly increases anxiety-like behavior, an effect which is blocked by acute fluoxetine treatment but not by the 5-HT_1A_ antagonist WAY 100,635 (Maximino et al., 2014c); WAY 100,635 was able to block the fear-induced analgesia caused by alarm substance (Maximino et al., 2014c), and low doses of this drug blocked the effect of alarm substance on behavior in the novel tank test (Nathan et al., 2015). Methysergide, a non-selective 5-HT receptor antagonist, also blocked the effects of alarm substance in the novel tank test (Nathan et al., 2015). A role for the 5-HT_1A_ receptor was also observed in the light/dark test, where antagonists show an anxiolytic-like effect (Maximino et al., 2013c), and in the novel tank test, where antagonists can either increase (Nowicki et al., 2014) or decrease (Maximino et al., 2013c) anxiety-like behavior. Finally, antagonists at the 5-HT_2_ and 5-HT_3_ receptors increase anxiety-like behavior in the novel tank test (Nowicki et al., 2014), while antagonists at the 5-HT_1B_ receptor decrease anxiety-like behavior in this test, but not in the light/dark test (Maximino et al., 2013c).

The cholinergic system is emerging as another important target for the pharmacological modulation of anxiety-like behavior in zebrafish. The acetylcholinesterase inhibitor physostigmine has been shown to decrease bottom-dwelling in the novel tank test, an effect consistent with reduced anxiety (Cho et al., 2012). Nicotine, an antagonist at nicotinic cholinergic receptors, produces ample and consistent anxiolytic-like responses in the novel tank test (Levin et al., 2007), while in the aquatic plus-maze no effect was observed (Sackerman et al., 2010). The effects of nicotine in the novel tank test are mediated by the α_7_ and α_4_β_2_ nicotinic receptors, as antagonists for these receptors block the anxiolytic-like effects of nicotine (Bencan and Levin, 2008).

Adenosine and its receptors were implicated in the pathogenesis of anxiety-like behavior (Ruby et al., 2011). Caffeine is a non-selective adenosine rececptor antagonist, and has consistently been shown to increase anxiety-like behavior in zebrafish (Egan et al., 2009; Wong et al., 2010; Maximino et al., 2011a,b). These effects of caffeine on the light/dark test are mimicked by drugs which block the adenosine A_1_ receptor, but not A_2_ receptors, suggesting a participation of the first, but not the latter, in zebrafish anxiety (Maximino et al., 2011a). Interestingly, the A_1_ receptor has also been shown to protect against the convulsive actions of pentylenotetrazole (Siebel et al., 2015), while both receptors have been implicated in the amnestic effects of scopolamine in the inhibitory avoidance test in zebrafish (Bortolotto et al., 2014). Treatment with IB-MECA, an agonist at A_3_ receptors (which so far have not been described in zebrafish) reduces dark preference in a nitric oxide- and serotonin-dependent manner, while the reduction of bottom-dwelling is dependent on nitric oxide but not serotonin (Maximino et al., 2014b).

These results suggest the potential of adult zebrafish for studying the mechanisms of anxiety-like behavior and discovering novel drug targets. In general, a good balance between demonstration of pharmacological isomorphism and seeking novel targets is seen in the zebrafish anxiety literature. Two other examples illustrate the potential of this species in describing the substrates of anxiety-like behavior.

In the first study, authors capitalized on the knowledge regarding the role of fibroblast growth factor (FGF) receptors on the development of the zebrafish brain (Thisse and Thisse, 2005). s*piegeldanio*, a mutant with reduced FGF_1A_ receptor function, was shown to have increased aggressive and exploratory behavior and decreased neophobia and anxiety (Norton et al., 2011). These animals show reduced dual specificity phosphatase enzyme *dusp6* and phosphorylated extracellular signal-regulated kinase in the inferior lobe of the hypothalamus (Norton et al., 2011). While a decreased expression of the isoform A of the serotonin transporter in the raphe is also observed, treatment with fluoxetine does not rescue the behavioral phenotype (Norton et al., 2011); instead, histamine N-methyltransferase is upregulated in the brains of *spiegeldanio* mutants, which show decreased histamine levels in the preoptic area and raphe nucleus (Norton et al., 2011). Indeed, treatment with the histamine N-methyltransferase inhibitor tacrine rescues not only the hypo-histaminergic profile but also the behavioral syndrome associated with reduced FGF1A receptor signaling (Norton et al., 2011).

In another study, knockdown of *otpa*, a gene which is duplicated in zebrafish in relation to vertebrates is used to circumvent the lethality of homozygous null mutations in mice (Amir-Zilberstein et al., 2012). OTP is a homeodomain protein that is highly expressed in the neuroendocrine hypothalamus (Blechman et al., 2007), suggesting a role in regulating stress responses. In zebrafish, two isoforms are present; null mutants for *otpa* show normal basal expression of CRF in the brain, but exposure to a stressor does not increase CRF expression in the mutants (Amir-Zilberstein et al., 2012). These animals also show less bottom-dwelling in the novel tank test (Amir-Zilberstein et al., 2012). A series of experiments demonstrated that Otp associates phosphorylated cAMP response element-binding protein (pCREB) to recruit the *crf* and *a2bp1* promoters in response to stressors; the latter promoter leads to the expression of a short variant of the PAC1 receptor for the pituitary adenylate cyclase-activating peptide (PACAP) (Amir-Zilberstein et al., 2012). Gene knockdown of the short variant of PAC1 leads to increased behavioral and CRF responses to stressor, and overexpression of the short form in *otpa*-positive neurons in the hypothalamus increases basal and stimulated CRF expression (Amir-Zilberstein et al., 2012).

PACAP and the PAC1 receptor have been implicated in post-traumatic stress disorder (Ressler et al., 2011). An interesting correlation with those results is the observation of the behavioral and neurochemical effects of PACAP intracerebroventricular (i.c.v.) injections in goldfish, a closely-related cyprinid (Maruyama et al., 2006). This peptide suppresses food intake and induces a significant increase in the expression of *crf* mRNA; both effects are blocked by an CRF_1_ receptor antagonist (Matsuda et al., 2006a). Moreover, PACAP also decreases locomotion in goldfish, albeit at a concentration higher than that needed to produce an anorexigenic effect (Matsuda et al., 2006a). While this locomotor effect is not necessarily suggestive of stress, an important research program has emerged on the role of hypothalamic neuropeptides in stress and feeding responses in goldfish (Matsuda, 2009; Matsuda et al., 2011a).

**Figure 2:**
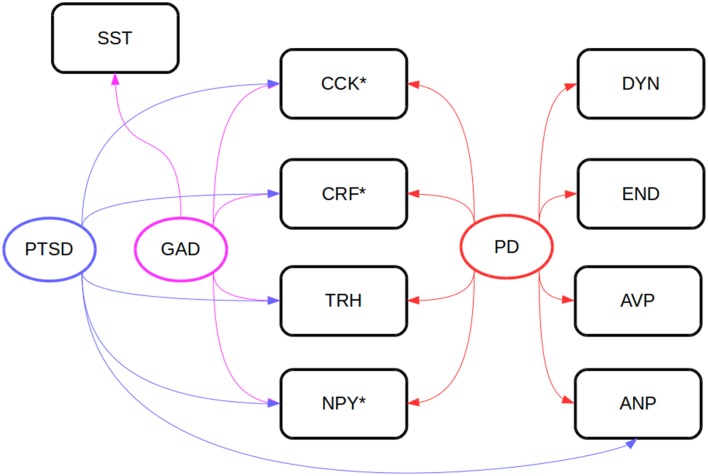
**Neuropeptides involved in anxiety disorder in clinical samples (Steckler, 2008)**. Peptides marked with asteriks (^*^) have been investigated in goldfish (*Carassius auratus*) psychomotor activity, anxiety, or feeding assays. AVP, vasopressin; ANP, atrial natriuretic peptide; CCK, cholecystokinin; CRF, corticotropin-releasing factor; DYN, dynorphin; END, β-endorphin; GAD, generalized anxiety disorder; NPY, neuropeptide Y; PD, panic disorder; PTSD, post-traumatic stress disorder; SST, somatostatin.

Some hypothalamic regions responsible for stress responses are also involved in food intake, and orexigen and anorexigen peptides produced and secreted from those areas regulate feeding. Some regulatory peptides involved in the organization of energetic homeostasis, such as ghrelin, orexin, galanin, thyrotropin-releasing hormone, PACAP, vasoactive intestinal peptide (VIP), and CRF, modulate different types of behavior after central or peripheral administration in mammals and teleost fish (Matsuda, 2009; Matsuda et al., 2011a). Some of these peptides have been linked to psychiatric disorders in humans (Figure 2). The i.c.v. and intraperitoneal injections of PACAP and VIP inhibits feeding and locomotion in goldfish (Matsuda et al., 2006a); PACAP injections also increase the expression of CRF mRNA in the brain, an effect which is mimicked by excessive feeding (Maruyama et al., 2006). While CRF has been implicated in anxiety and stress (Takahashi, 2001), there is also some evidence for a role in appetite control. CRF injections in the brain, but not in the periphery, is also anorexinogenic in goldfish (De Pedro et al., 1993). However, CRF injections increase locomotion (Maruyama et al., 2006) and produce an anxiogenic-like effect in the novel tank test (Matsuda et al., 2013).

The central effects of CRF on feeding in goldfish are independent on cortisol, as injections of this hormone do not alter food intake (de Pedro et al., 1997). CRF receptors also mediate the anorexinogenic effects of α-melanocyte stimulating hormone (α-MSH), since CRF antagonists block the effects of i.c.v. α-MSH injections on feeding but α-MSH antagonists do not block the effects of i.c.v. CRF (Matsuda et al., 2008). Subsequent studies demonstrated that the anorexigenic actions of both CRF and α-MSH are blocked by a gonadotrophin-releasing hormone (GnRH) type I receptor antagonist, suggesting that these stress peptides induce the release of GnRH2 which ultimately mediates their effects on feeding (Kang et al., 2011a).

Other neuropeptides involved in feeding have been shown to modulate locomotor activity and/or anxiety in goldfish. I.c.v. injections of ghrelin increase locomotion, while intraperitoneal injections decrease it; the intraperitoneal injection is also unable to alter dark preference (Kang et al., 2011c). Further studies using an acylated peptide demonstrated that this isoform increases food intake and increases locomotor activity when injected i.c.v. or intraperitoneally, while des-acyl ghrelin has no effect on locomotion, suggesting that the acylation is necessary for the psychomotor effects of ghrelin (Matsuda et al., 2006b).

The hypocretin/orexin system has been implicated in zebrafish feeding and arousal (Volkoff and Peter, 2006; Chiu and Prober, 2013). Orexin expression is upregulated in the goldfish hypothalamus after food deprivation and downregulated by intraperitoneal glucose injections (Nakamachi et al., 2006). I.c.v. injection of orexin A, but not orexin B, increases food intake in goldfish, while an anti-orexin antibody decreases food intake (Nakamachi et al., 2006). Orexin A, but not orexin B or an anti-orexin antibody, increases locomotion (Nakamachi et al., 2006), dark preference, and bottom-dwelling (Nakamachi et al., 2014) when injected i.c.v.; these effect are blocked by pre-treatment with a OX1R antagonist (Nakamachi et al., 2014).

Cholecystokinin (CCK) is a gut polypeptide involved in stimulating the digestion of fat and protein. Post-translational modifications produce hormones with variable number of amino acids, including CCK4 (which acts primarily in the central nervous system, with little effect on the gastrointestinal tract) and CCK8 (which acts both centrally and peripherally) (Fink et al., 1998). In goldfish, i.c.v. CCK8 administration is anorexigenic and increases *pomc* mRNA levels, but not *crf*, in the brain (Kang et al., 2010). Pretreatment with CRF antagonists block this anorexigenic effect of CCK8 (Kang et al., 2010). Intraperitoneal CCK8 also produces an anorexigenic effect (Kang et al., 2010) that is blocked by pretreatment with the NMDA receptor antagonist MK-801 (Kang et al., 2011b).

Neuropeptide Y (NPY) has been implicated in feeding and, recently, has been identified as a potential target for the treatment of anxiety disorders (Garner et al., 2009). I.c.v. injection of NPY reduces locomotor activity and dark preference in goldfish (Matsuda et al., 2011a) and increase food intake (López-Patiño et al., 1999; Miura et al., 2006). The orexigenic effect of NPY is blocked by pretreatment with Y_1_ receptor antagonists (López-Patiño et al., 1999; Miura et al., 2006), while the anxiolytic-like effect is not (Matsuda et al., 2011a). However, the injection of an Y_4_ receptor antagonist mimicks the effects of NPY (Matsuda et al., 2011a), suggesting that feeding and anxiety are mediated by different mechanisms in the goldfish brain. Interestingly, chronic fluoxetine treatment increases *npy* expression in the zebrafish brain (Wong et al., 2013).

In addition to feeding-related peptides, stress-related peptides were also tested in goldfish for their effects on stress, locomotion, and feeding. Octadecaneuropeptide is an endozepine which acts as an agonist at the translocator protein 18 kDa (formerly known as peripheral benzodiazepine receptor) and at a metabotropic receptor and an inverse agonist at benzodiazepine binding sites at central GABA_A_ receptors (Gamier et al., 1994). I.c.v. ODN administration increases locomotor activity and dark preference in goldfish, an effect which is blocked by pretreatment with benzodiazepine antagonists, but not by antagonists at the metabotropic receptor, suggesting that its anxiogenic-like and locomotor effects are mediated by the benzodiazepine binding site (Matsuda et al., 2011b). ODN and derivative peptides also inhibit food intake in this species, an effect which is blocked by metabotropic receptor antagonists but not flumazenil, suggesting mediation by the metabotropic receptor but not by the benzodiazepine binding site (Matsuda et al., 2007). Interestingly, i.c.v. ODN injection increases proopiomelanocortin (*pomc*) mRNA levels, but not *crf*, and its anorexigenic effects are blocked by pretreatment with either a α-MSH antagonist or a CRF antagonist (Matsuda et al., 2010).

#### Aversive conditioning in goldfish

In addition to these experiments analyzing the behavioral effects of neuropeptides in goldfish, other experiments also analyzed learning and memory functions of specific brain regions. These experiments capitalize on the increased brain size of goldfish in relation to zebrafish, making ablation experiments and electrode implantation much easier in the first than in the latter cyprinid (Table 1). While functional considerations are not normally used to establish homology claims, these experiments were crucial in advancing a second wave of discussion regarding the limbic telecephalon of teleost fish (Butler and Hodos, 2005).

The amygdaloid nuclei possess a variety of neurobiological roles, among which its participation in emotional behavior and learning are intensely studied (LeDoux, 2012). Some amygdaloid nuclei, including the basolateral portions (BLA), have been demonstrated to be central for avoidance learning in rodents (Choi et al., 2010; Lázaro-Muñoz et al., 2011). In this form of instrumental conditioning, the animal associates a response (usually shuttling in a box) with avoidance of an aversive consequence, such as an electric shock, that has been signaled by a sound or light stimulus. Two-factor theory proposes that, in avoidance conditioning, the subject first learns that the signaling stimulus predicts the aversive stimulus (Pavlovian conditioning) and then learns that a particular behavior (avoidance) causes termination of both the signal and the aversive stimulus (operant conditioning) (Maia, 2010). In this sense, the conditioned fear to the signal drives learning of the avoidance response.

The medial pallium (MP) of teleost fish has been proposed to be homologous to the mammalian BLA (Maximino et al., 2013b). Interestingly, lesions in this region, but not in the lateral pallium (LP) impair two-way avoidance learning in goldfish (Portavella et al., 2002, 2004a,b; Portavella and Vargas, 2005; Vargas et al., 2012). Interestingly, the same region shows increased *cfos* expression in zebrafish after exposure to the light/dark test (Lau et al., 2011; von Trotha et al., 2014). LP lesions, on the other hand, impair spatial learning, but not active avoidance conditioning, except when a time interval is added between the signal and the electric shock (“trace conditioning”) (Portavella et al., 2002; Portavella and Vargas, 2005).

As is the case with avoidance learning, the medial pallium is also involved in conditioned taste aversion (CTA), which involves the tendency to avoid the ingestion of substances that were previously associated with visceral discomfort. CTA is impaired by BLA lesions in rodents (Reilly and Bornovalova, 2005), and is characterized by long interstimulus intervals, high stimulus specificity, and fast acquisition—in fact, CTA can be established by a single trial (Garcia et al., 1955). The *medial pallium*, especially at precommissural levels, receives gustatory information from the diencephalic tertiary gustatory nucleus in the Rainbow trout *Onchorynchus mykiss* (Folgueira et al., 2003), althought there is no evidence that general visceral information reach this region (Yoshimoto and Yamamoto, 2010). Goldfish are able to successfully avoid a gustatory stimulus which was followed by lithium chloride; whole-telencephalon ablation and lesions in the medial pallium, but not in the lateral pallium or cerebellum, impair the acquisition of CTA in this species (Martín et al., 2011). Interestingly, when the gustatory stimulus was paired with an electrical shock, a conditioned bradycardia develops that is not impaired by telencephalic ablation (Martín et al., 2011).

The pharmacological or neurochemical bases of these effects are unknown. However, recent experiments using microinjection of drugs in the telencephalon suggest a role for the glutamate-nitric oxide pathway in that process (Xu et al., 2003, 2009). Goldfish were trained in an active avoidance paradigm similar to that proposed by Portavella and colleagues (Vargas et al., 2012). Escape responses were defined as shuttling made after the onset of both the light signal and the electric shock, while avoidance responses were made after onset of light signal but before the electric shock. The injection of D-AP5, an antagonist at glutamatergic *N*-methyl-D-aspartate (NMDA) receptors, before training did not alter the performance of escape responses, but significantly impaired the acquisition of avoidance responses (Xu et al., 2003). When this drug was injected after training, no amnesic effect was observed, suggesting a participation of this receptor in the acquisition phase, but not in the consolidation of the aversive memory (Xu et al., 2003). In a second set of experiments, Xu et al. (2009) injected nitric oxide synthase or guanylate cyclase inhibitors, impairing the production of nitric oxide or cyclic guanosine monophospate (cGMP), respectively. Injection of these drugs before training impaired the acquisition of avoidance responses, without effects on escape responses. Interestingly, microinjection of these drugs in the medial pallium imediately after training also impaired avoidance responses, suggesting a participation of the nitric oxide-cGMP system on the consolidation of the active avoidance memory (Xu et al., 2009).

Both microinjection and ablation experiments underline the main advantage of goldfish in relation to zebrafish (Table 1): while zebrafish certainly has important advantages from the point of view of genetics and molecular toolboxes, the size of goldfish allows more easily for “classical” neuroscience techniques (drug microinjection, structure lesion, *in vivo* electrophysiological recordings). Indeed, while microinjection techniques are being introduced in zebrafish (Kizil and Brand, 2011; Barbosa et al., 2012), they are much easier to make in an animal with a bigger brain and body. Thus, experiments with goldfish could complement results found in zebrafish, especially regarding localized interventions.

#### Aggression in the Siamese fighting-fish

In aggression research, the Siamese fighting-fish *Betta splendens* was widely used during the 1970s and 1980s due to the well-described characteristics of the appetitive and consummatory aspects of its aggressive behavior (Simpson, 1968). Moreover, as is the case with other ornamental fish species, maintenance, and housing costs are low, and keeping fighting-fish in laboratories is relatively simple (Clotfelter et al., 2007; Kania et al., 2012). Moreover, a stereotaxic atlas of the fighting-fish telencephalon has been constructed, facilitating the description of neural systems underlying behavior (Marino-Neto and Sabbatini, 1988).

*Betta splendens* usually exhibit salient aggressive behavior toward conspecifics, including a characteristic appetitive element—the aggressive display –, which is characterized by the extension of operculae, extension of medial, and caudal fins, and an intensification of body color (Simpson, 1968). When the adversary counter-displays, an escalation ensues, resulting in attacks that include offensive biting (Bronstein, 1983, 1994). *Betta* males also present aggressive display when exposed to a mirror (Miley and Burack, 1977); mirror presentation can therefore be used in lieu of a conspecific since it can simulate and aggressive encounter without harming the animals, since no difference between the strength of aggressive display toward a mirror or a conspecific (Miley and Burack, 1977).

While studying the aggressive display is useful for analyzing aggression, other techniques can also be used to assess the level of aggressive motivation in fighting-fish. Tapping on the classical studies of rodent motivation using runways, some studies analyzed the aggressive readiness by conditioning fighting-fish to swim through an “aquatic runway” in order to gain access to a conspecific or a mirror stimulus. It has been demonstrated that the level of aggressive motivation (as assessed by the time taken to reach the target area) is associated with combat readiness (number of displays) (Hogan and Bols, 1980). “Social reinforcement”—that is, instrumental behavior controlled by the opportunity for aggressive displays—has been used for a wide variety of applications in fighting-fish; interestingly, *Betta splendens* display self-control for mirror access, choosing delayed access to a mirror stimulus with longer duration instead of immediate access to a short duration of mirror presentation (Collins, 2008). These observations could complement work on the zebrafish three-choice serial reaction time task (Parker et al., 2012, 2013a,b) to build a research program on impulsive control disorders.

There is some evidence that *Betta splendens* possess significant cerebral laterality—the division of cognitive functions between both brain sides—in relation to other anabantoid fish, given that they demonstrate an eye use preference in aggressive interactions (Clotfelter and Kuperberg, 2007). Lateralized individuals also present higher group cohesion and better performance in spatial tasks in relation to non-lateralized individuals (Clotfelter and Kuperberg, 2007).

Serotonin (5-HT) has been implicated in the modulation of aggressive behavior in different species (Takahashi et al., 2011), including fish (Herculano and Maximino, 2014). Acute treatment with low doses of fluoxetine decreases the duration of aggressive display (Lynn et al., 2007; Dzieweczynski and Hebert, 2012; Forsatkar et al., 2013), suggesting an inhibitory role for 5-HT in fighting-fish aggression. However, neither the 5-HT synthesis inhibitor *para*-chlorophenylalanine nor the 5-HT precursor L-tryptophan changed display behavior (Clotfelter et al., 2007), suggesting that phasic, but not tonic, 5-HT controls aggressive behavior. Consistently with that hypothesis, intramuscular acute injections of 5-HT and 8-OH-DPAT (an agonist at 5-HT_1A_ and 5-HT_7_ receptors) decrease the duration and readiness of aggressive displays, while the 5-HT_1A_ receptor antagonist did not produce an effect (Clotfelter et al., 2007). The effects of chronic treatment with fluoxetine are mixed, with some authors describing decreases in aggressive displays (Kania et al., 2012) while other authors described no effect (Clotfelter et al., 2007).

### Honeybees: Aversive control and impulsivity

Honeybees comprise the genus *Apis*, which comprises seven species and 44 subspecies (Martín et al., 2011). While distributed in the whole world, honeybees appear to have originated in South and Southeast Asia and Africa (Engel, 1999). The Western honey bee (*Apis mellifera*) had its genome fully sequenced in 2006 (http://hymenopteragenome.org/beebase/), and at least since the description of the waggle dance by Karl von Frisch it has been proposed as a model organism for ethology and comparative cognition (Smith et al., 2000; Whitfield et al., 2006). Moreover, as eusocial species, honeybees are increasingly being used to understand how the social environment can shape behavior, including social learning, predator cues, and social decision making (Menzel, 1983). A comparative overview of the advantages and disadvantages of using honeybees in neuroscience can be found in Table 2.

**Table 2:** **Advantages and disadvantages for *Drosophila melanogaster* and *Apis mellifera* as models in behavioral neuroscience**.

***Drosophila melanogaster* (Fruit fly)**	***Apis mellifera* (Honeybee)**
**GENERAL ADVANTAGES**	
•Model organism in developmental genetics •Rapid generation time •Cost effective/high density stocking •External development	•Model organism in social and cognitive neurosciences •Eusocial species with different body morphs associated with castes •External development
**IMAGING/NEUROANATOMY**	
•Small size ideal for microscopy •Compact neuronal network revealable by two-photon or confocal imaging •GAL4/UAS enhancer trapping for neuroanatomical determination and pharmacogenetic ablation •Broad homologies (“Urbilaterian brain”)	•Small size ideal for microscopy •Compact neuronal network revealable by two-photon or confocal imaging •Broad homologies (“Urbilaterian brain”)
**BEHAVIOR**	
•Well-characterized exploratory behavior •Some psychotropic drug (e.g., ethanol) effects characterized	•Ethological/naturalistic assays for social behavior •Aversive control assays (escape/avoidance, sting extension responses, classical conditioning) •Cognitive bias •Perception, learning and memory assays
**GENETIC/GENOMIC RESOURCES**	
•More than 1.5 million sequenced genes •More than 75,000 annotated gene expression patterns, including miRNAs •Transgenesis using retroviral and transposon vectors •Rapid mutagenesis (TILLING, ENU screens, insertional mutagenesis, zing finger nucleases, TALENs, CRISPR/Cas) •Large collection of naturally occurring and synthetic mutants •Transgenic/mutant outcrossing to wild-type populations relatively easy •Rapid gene knockdown using antisense morpholinos •Genome-wide association analysis	•More than 1 million sequenced genes •Description of single nucleotide polymorphisms by alignment with Africanized honey bee sequences •Smaller genome than *Drosophila* •Genes involved in circadian rhythms, RNA interference (RNAi) and DNA methylation more greater similar to vertebrate genomes than *Drosophila* and *Anopheles* genomes •Some miRNAs with to have caste- and stage-specific expression
**PHARMACOLOGY/PHYSIOLOGY**	
•Neuropathology models •Conservation of classic neurotransmitters (monoamines, amino acids)	•Conservation of classic neurotransmitters (monoamines, amino acids)
**DATABASES**	
•Flybase: http://flybase.org •Der Pylz: http://mushroombody.net	•BeeBase: http://hymenopteragenome.org/beebase/
**DISADVANTAGES**	
•Small size for tissue samples and microdialysis •Non-conserved physiology (e.g., open circulatory system) •Non-conserved CNS *bauplan* •No physiological techniques for awake, behaving animals •Important neurotransmitters and hormones not conserved in vertebrates (e.g., octopamine, ecdysteroid) •Few well-established behavioral assays	•Long generation times in relation to omodel organisms •Small breeding populations •Severe effect of inbreeding preclude the development of isogenic bee lines •Small size for tissue samples and microdialysis •Non-conserved physiology (e.g., open circulatory system) •Non-conserved CNS *bauplan* •No physiological techniques for awake, behaving animals •Important neurotransmitters and hormones not conserved in vertebrates (e.g., octopamine, ecdysteroid)

Honeybees, especially *Apis mellifera*, are increasingly demonstrating their potential as models in behavioral studies, following the inclusion of invertebrates in neurobehavioral research (Leadbeater and Chittka, 2007). In particular, honeybees are capable of complex decision making, presenting cognitive biases (Wilson-Sanders, 2011) and self-control (Bateson et al., 2011).

Honeybees have long been shown to be sensitive to aversive control (Hunt, 2007; Curran and Chalasani, 2012). Abramson (1986) demonstrated that honeybees quickly acquire aversive control in punishment, escape, and avoidance contingencies when an aversive odor (formic acid) is used. In a semi-naturalistic setting, honeybees trained to discriminated between two differently colored targets quickly acquire avoidance responses when response to one of the targets is associated with an electric shock in the proboscis (Abramson, 1986). Honeybees also present a sting extension reflex, which is exhibited when the animal is subjected to noxious stimuli; this reflex can be conditioned so that bees learn to extend their sting in response to odorants previously paired with an electric shock (Vergoz et al., 2007; Tedjakumala and Giurfa, 2013). Interestingly, dopamine and 5-HT (but not octopamine or 20-hydroxyecdisone) have been shown to decrease the conditioned sting extenstion response, while 5-HT_2_ receptor antagonists increase responsiveness (Vergoz et al., 2007; Tedjakumala et al., 2014).

While these results suggest a conserved role for specific 5-HT receptors in simple aversive control, other, more complex phenotypes can also be observed. In an interesting set of experiments, Bateson et al. (2011) first trained honeybees to associate a two-component odor mixture with either a reward (sucrose solution), punishment (quinine solution) or a less valuable reward (diluted sucrose solution); after training, animals were presented with unreinforced (test) trials in which three different odors mixtures, with intermediate concentrations of the mixture of the original compounds, were presented in addition to the two original odors. Animals responded to the original odor associated with the reward by extending the proboscis, while the odor that was associated with punishment or a less rewarding consequence did not elicit proboscis extension. Intermediate concentrations of the two-odor mixtures produced a mixed response, with mixtures with a higher concentration of the punishment-associated odors eliciting less proboscis extension responses. Interestingly, when animals were vigorously shaken for 60 s before testing, responding toward these concentrations was further decreased, suggesting a “cognitive bias”—that is, agitated honeybees classified ambiguous stimuli as predicting punishment (Bateson et al., 2011). Moreover, shaken bees showed decreased levels of dopamine, octopamine and serotonin in the hemolymph (Bateson et al., 2011). The authors suggested that this demonstration of a state-dependent modulation of categorization in honeybees has more in common with vertebrate behavior than previously thought.

Given that negative affective states such as anxiety and depression are associated with increased punishment expectancy, greater attention to potential threats, and a tendency to interpret ambiguous stimuli as threats, this “cognitive bias” demonstrates that this behavior can be used to model some aspects of psychiatric disorders. The neural mechanisms underlying negative cognitive biases are so far unexplored, but the observation that both dopamine and serotonin are diminished suggests similarities with the mechanisms underlying aversive control. However, this study also demonstrated a decrease in circulating octopamine levels in agitated bees—an effect which is difficult to reconcile with findings in vertebrates, since octopamine is found only in invertebrates. Moreover, in vertebrates the role of 5-HT on aversive control is highly dependent on receptor subtype and site of action, with 5-HT_2_ receptors increasing aversive responsiveness in the amygdala and decreasing it in the periaqueductal gray (Maximino, 2012; Zangrossi and Graeff, 2014).

In addition to aversive control and cognitive bias, important experiments demonstrated that honeybees are able to self-regulate their behavioral choices and make an economic choice for a delayed and bigger reward in opposition to an immediate small reward (Cheng et al., 2002). Experiments showing the capacity for “self-control” are important to understand impulsive choice, sometimes indexed by an alteration in the optimal delay discounting behavior described above (Arce and Santisteban, 2006). Cheng et al. (2002) described an experiment in which honeybees were trained to choose between a delayed sweet reward and an immediate less sweet reward, choosing the first over the latter. Food deprivation increases impulsive choice and brain dopamine levels (Mayack and Naug, 2015). In addition, successive negative contrast has been demonstrated in bumble bees (*Bombus impatiens*)—that is, animals adjust their choice toward less effort when the reward value is downshifted (Waldron et al., 2005). Although pharmacological, genetic, and biochemical experiments are still much needed to elucidate the isomorphism of these responses to vertebrate systems, they point to an exciting possibility of using bees to study impulse control and its social modulation.

### Fruit fly exploratory behavior and aggression

*Drosophila melanogaster* are widely used in different fields of the biomedical sciences, especially in genetics (Ankeny and Leonelli, 2011). *Drosophila* share the broad actions of essential neurochemical substrates (specific receptors, signaling enzymes and proteins, neurotransmitters systems) that are involved in emotional behavior (Schafer, 2002; Iliadi, 2009). Genetic techniques produced *Drosophila* mutants for genes associated with neurodegenerative disorders, making the species suitable for studying the pathological bases of these diseases (Muqit and Feany, 2002). These advances were made possible by the characteristics of fruit flies which turned them into a central model organisms in genetics (Table 2): low maintenance cost, short generation time (c. 2 weeks), high fertility, and, of course, the availability of a research community and an extensive toolbox to manipulate gene expression in this species (Muqit and Feany, 2002; Schafer, 2002; van Alphen and van Swinderen, 2013).

As is the case with most model organisms, the bottleneck for its introduction in the behavioral neurosciences was the availability of neurobehavioral assays (Iliadi, 2009; van Alphen and van Swinderen, 2013). Insects such as *Drosophila* and honeybees exhibit some defensive behaviors which can be interpreted as anxiety-like and/or fear-like. In *Drosophila*, centrophobism/thigmotaxis has been proposed to represent anxiety- or fear-like behavior (Besson and Martin, 2004; Iliadi, 2009).

Thigmotaxis was first observed in *Drosophila* as an after-effect of diethylether anesthesia, although it is present at basal levels in non-treated flies (Götz and Biesinger, 1985). It was observed that, when exploring a novel circular arena, flies avoid the arena center, an effect which is exacerbated after anesthesia (Götz and Biesinger, 1985). It was later observed that the preference for the arena boundaries is not controlled only by center avoidance (centrophobism) or wall preference due to tactile stimulation (thigmotaxis), and that flies prefer sheter-like environments (alcoves or dark corners), but only after the initial boundary exploration waned (Soibam et al., 2012). In both cases, an explicit “wall-following” is controlled by a complex combination of variables, including exploratory behavior (“curiosity”-driven) and avoidance due to the novelty of the environment. This is consistent with Montgomery's (1954) proposal that both approach and avoidance control exploratory behavior, the basis for the use of exploratory behavior in rodent models of anxiety.

This “wall-following” behavior is controlled by the mushroom bodies (MBs), one of the most well-studied central brain-like structures in the *Drosophila* brain. Hydroxyurea ablation of the MBs diminishes wall-following behavior, an effect that is replicated by genetic disruption of the synaptic transmission in γ lobes, but not in α/β lobes (Besson and Martin, 2004). Mutations that affect the cyclic adenosine monophosphaste (cAMP) pathway also decrease centrophobism, suggesting a participation of the cAMP-PKA pathway in that behavior (Lebreton and Martin, 2009). Finally, it has been shown that neurons expressing a substance P-like peptide in the fan-shaped body of the central complex are involved in wall-following, as genetic ablation of these cells increases this behavior (Kahsai et al., 2010).

These results highlight an important feature of *Drosophila* research: due to the genetic tractability of the model, manipulations which the use of Gal4/UAS lines to drive gene expression in a cell-specific manner (Aso et al., 2009). These powerful techniques, which are increasingly being used in zebrafish (Scott et al., 2007), allow for the expression of photosensitive proteins such as channelrhodopsin and halorhodopsin, as well as proteins such as tetanus toxin light chain (TeTxLC) or KillerRed in zebrafish or the temperature-sensitive protein Shibire in *Drosophila*, to inactivate a specific circuit or cell type expressed in a specific region. These powerful tools are increasingly being used in circuit neuroscience in genetically tractable organisms. On the other hand, the “allure” of high-technology research produces a tendency, in such organisms, for fundamental “low-tech” research to be ignored. In the case of *Drosophila* wall-following behavior, basic pharmacological research using clinically effective drugs has not yet been made, and therefore wall-following/thigmotaxis/centrophobism lacks pharmacological isomorphism.

Another important set of behaviors which have been studied in *Drosophila* and have consequences for biological psychiatry is aggression (Chen et al., 2002; Iliadi, 2009; van Alphen and van Swinderen, 2013). As is the case with most vertebrate species, fly aggressive behavior follows a pattern which includes behaviors without physical contact with the opponent, such as wing threat displays, and actions with direct physical contact, including fencing, holding, boxing, and tussling (Chen et al., 2002; Zwarts et al., 2012). Wing threat displays are threatening postures directed toward other males before fat charges. It is not known whether these display postures, as appetitive elements, represent an emotion such as anger (Iliadi, 2009).

Serotonin has been implicated in the control of aggressive behavior in vertebrates (Miczek et al., 2007; Carrillo et al., 2009). In *Drosophila*, the role of 5-HT in aggression is unclear, as 5-HT treatment does not alter aggression (Baier et al., 2002), and artificial selection for discordant levels of aggressive behavior does not alter the expression of genes involved in serotonergic signaling (Dierick and Greenspan, 2006). Nonetheless, pharmacological or genetic elevation of the serotonergic tonus increases aggression, and genetic silencing of 5-HTergic circuits blocks the effects of pharmacological induction while sparing aggression (Dierick and Greenspan, 2007). Expression of temperature-sensitive dTrpA1 channels in 5-HTergic neurons allows for the acute activation of these cells, accelerating the escalation of fights (Alekseyenko et al., 2010). In general, then, acute increases in 5-HT levels increase aggression, but it is not known whether this mechanism is present physiologically.

### Reptile neuroethology

While zebrafish and *Drosophila* are important reference species mainly due to their usefulness as model organisms, reptiles are important due to their position in the vertebrate phylogeny; reptile species are positioned at the anamniote-amniote transition (Figure 1), representing an important evolutionary junction that is mostly underrepresented in behavioral neuroscience.

Among reptile species, anole lizards are among the most widely studied in evolutionary ecology and ethology (Greenberg, 2002; Lovern et al., 2004). The genus *Anolis* is a diverse and widespread New World taxon that includes the green anole *Anolis carolinensis*, a small, diurnal, insectivorous lizard from the United States southeast that is convenient to observe and easy to maintain in the laboratory (Greenberg, 2002). Behavioral inventories (ethograms) that emphasize social dynamics have been produced for anole lizards (Greenberg and Noble, 1974; Greenberg, 1977) and other reptiles. Antipredator behavior has also been described in different lizard species, and include tonic immobility (Edson and Gallup, 1972; Hennig, 1977, 1979; Santos et al., 2010; Maximino et al., 2014a), flight (Hennig, 1979; Maximino et al., 2014a), modifications of exploratory behavior (Greenberg, 2002) and of refuge use (López et al., 2005). The advantages of using small reptiles as laboratory models (Table 3) include its high availability in the wild (allowing for the establishment of laboratory colonies which are continuously replenished with wild stock to obtain field-relevant laboratory studies), the variety of life history traits between species, their close phylogenetic relationship with birds (forming the most basal extant amniotes), and ease of maintenance and cost-effectiveness without sacrificing ecological relevance (Lovern et al., 2004). Moreover, brain atlases are also present for *A. carolinensis* (Greenberg, 1982), *Gallotia galloti* (Del Corral et al., 1990) and *Gekko gekko* (Wang et al., 2008).

**Table 3:** **Advantages and disadvantages for *Anolis carolinensis* and *Gallus gallus* as models in behavioral neuroscience**.

***Anolis carolinensis* (Carolina anole)**	***Gallus gallus* (Chicken)**
**GENERAL ADVANTAGES**	
•Easily obtainable from the field; allows for replenishment of lab colonies with wild stocks •Easy maintenance of naturalistic habitats in the laboratory •Phylogenetic position in the amniote-anamniote transition	•Readily avaliable •Surgical manipulations and morphogen injection *in ovo* •Relatively easy to establish *in vitro* cultures •Phylogenetic position in the amniote-anamniote transition
**IMAGING/NEUROANATOMY**	
•Conservation of most brain regions (basal ganglia, amygdaloid nuclei, hippocampus, hypothalamus, isocortex) •Stereotaxic atlas useful for lesion/stimulation studies •Small adult brain size allows reduced number of sections for histological analysis	•Conservation of most brain regions (basal ganglia, amygdaloid nuclei, hippocampus, hypothalamus, isocortex)
**BEHAVIOR**	
•Stress responses and social (courtship/aggressive) behavior well-characterized	•Distress vocalizations and sleep patterns well-characterized
**GENETIC/GENOMIC RESOURCES**	
•More than 59,000 transcripts •More than 20,000 annotated gene expression patterns	•Over 1500 QTL mapped •Rapid gene knockdown using antisense morpholinos •Large collection of mutants and inbred strains •Transgenesis using retroviral vector and embryonic stem cells
**PHARMACOLOGY/PHYSIOLOGY**	
•Conservation of classic neurotransmitters (monoamines, amino acids) •Conservation of most neuropeptides (e.g., ACTH, CRF) •Conservation of immediate early genes (e.g., *cfos, jun, homer*)	•Conservation of classic neurotransmitters (monoamines, amino acids) •Conservation of most neuropeptides (e.g., ACTH, CRF)
**DATABASES**	
•Lizardbase: http://lizardbase.org/pages/index.html	•*Gallus* Genome Gbrowse: http://128.175.126.109/cgi-bin/gbrowse/gallus/ •GEISHA: http://geisha.arizona.edu/geisha/
**DISADVANTAGES**	
•No inbred strains •No transgenesis or knockdown technologies reported so far •Classical electrophysiological tools not well-developed; no functional MRI •Specifics of egg injection not readily transferable from chicks	

Socially submissive green anoles show conspicuous physiological and behavioral alterations at both the immediate (catecholamine surges, increased plasmatic levels of corticosterone and α-melanocyte-stimulating hormone (MSH), increased serotonergic activity in the midbrain, hindbrain, hippocampus and nucleus accumbens, body color alterations) and the long term (reduced androgen tone, elevated corticosterone and α-MSH levels, decreased dopaminergic activity in the hindbrain and midbrain, decreased courtship and perch selection behavior). While the endocrinology of social stress responses is well-established in *Anolis* and other reptile species, a more careful observation of behavioral alterations is still lacking (Greenberg, 2002; Summers et al., 2003; Øverli et al., 2007).

Lizards are prey to many predators and therefore are subject to intense predation pressure, which is largely responsible for the development of multiple defensive strategies (Greenberg, 2002; López et al., 2005; Thaker et al., 2009). Leal and Rodríguez-Robles (1995, 1997) analyzed antipredatory responses in two different anole lizard species (*Anolis cristatellus* and *Anolis cuvieri*) during encounters with its natural predator, the snake *Alsophis portoricensis*, and observed 13 different behavioral responses, of which approximately half are also involved in social interactions in these species. A stereotypical sequence of behavioral acts was observed, depending on the phases of predator-prey interaction. Understanding the underlying mechanisms which organize these behavioral displays is essential to systematization and characterization of defensive responses these animals, as well to facilitate comparative studies relating to the establishment of animal models.

As an example, Machado et al. (2007) analyzed flight responses of *Tropidurus montanus*, another squamate lizard, to feigned attacks. The experimenters observed that maximum flight distance did not differ between the sexes; the presence of a neighbor did not affect flight for males; and that males with neighbors maximized the time of flight when compared to males without a neighbor. Moreover, body length was not predictive of flight behavior. During capture, *T. montanus* relied on attempted escapes, cloacal discharges, threat displays, tail breakage and tonic immobility (TI), in no particular order. TI duration is also increased in *A. carolinensis* by the presence of a simulated predator (Hennig, 1977) and is higher at shorter distances from the simulated predator (Hennig et al., 1976).

Hennig (1979) studied the effects of the physical environment, time in captivity, and distance between potential predator and prey on defensive behaviors *Anolis carolinensis*. In addition to TI duration, flight latency was also recorded in order to determine if environmental factors affect these defensive behaviors similarly. The results revealed that the immediate testing environment is more important when a potential predator was nearby, and housing is more important at greater distances and after the first few days of adaptation to the new conditions. TI seems to be responsive to both changes in testing environment and time in captivity, while flight latency was only sensitive to the type of housing environment during captivity.

Many variables such as those mentioned above can potentially change defense responses, but it remains unclear how individual animals from the same population, sex, age, reproductive status, and other similar conditions of risk and costs, differ in their antipredator behavior. López et al. (2005) suggested that the propensity to take risks in a homogeneous group of adult male Iberian rock lizards (*Lacerta monticola*) varies in a “shy–bold” continuum and observed than the possible source of variation in antipredator behavior might also be related to small subtle differences in morphology, body condition, and health.

Tonic immobility has been equated with thanatosis, a secondary defense mechanism in which the animal displays “death-feigning” in response to external stimuli. This peculiar defensive behavior is assumed by many different lizard species (Edson and Gallup, 1972; Leal and Rodríguez-Robles, 1995, 1997; Machado et al., 2007; Santos et al., 2010). Immobility responses to acute and intense threat have been proposed to be a part of acute stress responses (Bracha, 2004; Moskowitz, 2004) and peritraumatic tonic immobility is predictive of development of post-traumatic stress disorder (Maia et al., 2011; Pires and da Costa Maia, 2013). Thus, tonic immobility has a potential translational value for biological psychiatry. In that sense, Maximino et al. (2014a) demonstrated that the Brazilian wall lizard *Tropidurus oreadicus* has a well-organized pattern of defensive behaviors that emerge after tonic immobility: immediately following the cessation of TI lizards initiate either freezing or (more frequently) a pattern of flight behavior that, in a circular arena, is characterized as “circling”; after that, animals return to careful exploration of the environment, employing thigmotaxis/centrophobic behavior and tongue-flicking (Maximino et al., 2014a). Importantly, these authors observed that this pattern is amenable to pharmacological dissociation, with panicolytic drugs (alprazolam, imipramine) decreasing TI duration and post-TI freezing or circling, but not exploratory (risk assessment-like) behavior, while anxiolytic drugs (diazepam) increased tongue-flicking and decreased thigmotaxis (Maximino et al., 2014a).

A careful consideration of the different antipredator behavioral strategies employed by different lizard species in naturalistic, semi-naturalistic, or experimental settings suggest that, as is the case with mammals, reptiles adjust their behavior in relation to “predatory imminence continua” (Fanselow and Lester, 1988). In safe environments such as a nest or a burrow (low predatory imminence), animals do not exhibit defensive behaviors, resuming their normal activity. In a novel environment, predatory imminence increases because the probability of encountering a predator increases, and the animal engages pre-encounter defensive behaviors—including thigmotaxis, tongue-flicking, air-licking, and posture changes—and body color changes associated with crypsis. When a threat is present, predator imminence increase further and the animal engages in post-encounter defensive behaviors (freezing). If post-encounter defensive strategies have failed (i.e., during contact with the predator), the animal switches to circa-strike defensive behaviors (flight/threat, tonic immobility, body thrashing, tongue-bunch, tail autotomy). These sequences have been observed in lizards both in the wild (Machado et al., 2007) and in laboratory environments (Hennig et al., 1976; Leal and Rodríguez-Robles, 1995, 1997; Maximino et al., 2014a), and have consequences for the development of novel models of anxiety disorders.

### The chick separation stress model

As is the case with other model organisms in developmental biology, laboratory chicks are used for a variety of reasons: availability and low price of fertilized eggs all the year round; fast development, with a duration similar to that of the mouse (21 days); absence of placenta and therefore of maternal effects after egg laying; and well-described developmental genetics (Table 3).

The chick separation stress paradigm exploits the strong attachment response of neonate fowl (*Gallus gallus*) and its typical distress vocalizations (DVoc) when socially isolated to model separation anxiety (Panksepp, 2011). Social separation initially produces an anxiety-like phase with high rates of DVoc, peaking in the first 3 min, followed by a decrease to about 50% of the initial rate within 10–25 min (Feltenstein et al., 2004; Sufka et al., 2006). While isolation for 3 min also produces stress-induced analgesia, hypothermia and ventral recumbency posturing (Sufka and Weed, 1994), the first effect is primarily mediated by novelty and not social isolation (Feltenstein et al., 2002). These phases can be pharmacologically dissociated, with anti-panic compounds (benzodiazepines, imipramine, clonidine, meprobamate, pentobarbital) attenuating DVocs in the first phase and antidepressant compounds (imipramine, maprotiline, fluoxetine) attenuating the second phase (Feltenstein et al., 2004; Sufka et al., 2006; Warnick et al., 2009). Moreover, social separation also increases plasmatic corticosterone levels (Feltenstein et al., 2003; Sufka et al., 2006). Interestingly, plasma corticosterone levels are higher when animals are isolated for 5–15 min, the period in which the first phase peaks, and this response is attenuated by isolation periods longer than 20 min (Sufka et al., 2006). Dopamine metabolism is increased in the telencephalon and diencephalon after 30 min (Hamasu et al., 2012). Interleukin-6, on the other hand, is elevated only by isolation for 120 min (Warnick et al., 2009). Chicks socially isolated for 5 min also show a “cognitive bias” in which they show higher responsiveness to aversive ambiguous cues, while animals isolated for 60 min are more responsive to both aversive and appetitive ambiguous cues (Salmeto et al., 2011); both forms of cognitive bias are attenuated by imipramine treatment, while clonidine did not alter responsiveness to any cue (Hymel and Sufka, 2012).

The amount of effort directed to the pharmacological and construct validation of the chick separation stress model is not a coincidence, as this test has mainly been used as a biobehavioral assay (Willner, 1991) to identify pharmacological compounds. In this sense, at least two target systems have been evaluated successfully. A participation of the opioidergic system was suggested by rodent social behavior assays (Burgdorf et al., 2011); in chicks, a participation of μ-opioid receptors is suggested by the observation that an agonist, DAMGO, attenuates DVocs in a 3 min isolation, while drugs acting at δ, κ or orphanin receptors are not effective (Warnick et al., 2005); the opposite effect is not observed by treatment with the non-selective opioid antagonist naloxone or the μ-opioid receptor antagonist CTOP, suggesting a phasic rather than tonic modulation. A role for NMDA receptors is suggested by a series of experiments in which intracerebroventricular injection of L-cysteine (Yamane et al., 2009a), glutathione (Yamane et al., 2007) and NMDA (Yamane et al., 2009b) blocks DVocs during a 10 min isolation test; in addition to this effect, these drugs also induce hypnotic effects. These results are surprising, since activation of the NMDA receptor induces anxiety- and panic-like behavior in rodents (Bergink et al., 2004) and fish (Herculano et al., 2015), and has been suggested to be mediated by NMDA-evoked GABA release.

In general, the chick separation stress test has proved to be an interesting behavioral assay to screen for potential anti-panic and antidepressive drugs, being able to detect hits and dismiss drugs which produce false positives in other tests (Sufka et al., 2006; Warnick et al., 2009). Moreover, the neuroendocrine markers associated with stress follow the timecourse expected for acute stress, although their projection to anxiety and mood disorders is still blurred. While very little is known about the brain structures which mediate behavior in this model, the avian brain is well-described (The Avian Brain Nomenclature Consortium, 2005), and *Gallus* is a traditional model organism in developmental biology (Fields and Johnston, 2005). Thus, as is the case with zebrafish (Norton, 2013; Stewart et al., 2014b), *Gallus gallus* is well-positioned to produce models in developmental psychopathology. The case of the chick separation stress test is particularly interesting from this point of view, since about one third of adult patients with separation anxiety disorder developed it during their childhood (Black and Grant, 2014) and infant behavioral inhibition, a temperamental trait that is characterized by high reactivity to environmental and social novelty, is predictive of adult anxiety disorders (Fox et al., 2005).

## Conclusions

**Figure 3:**
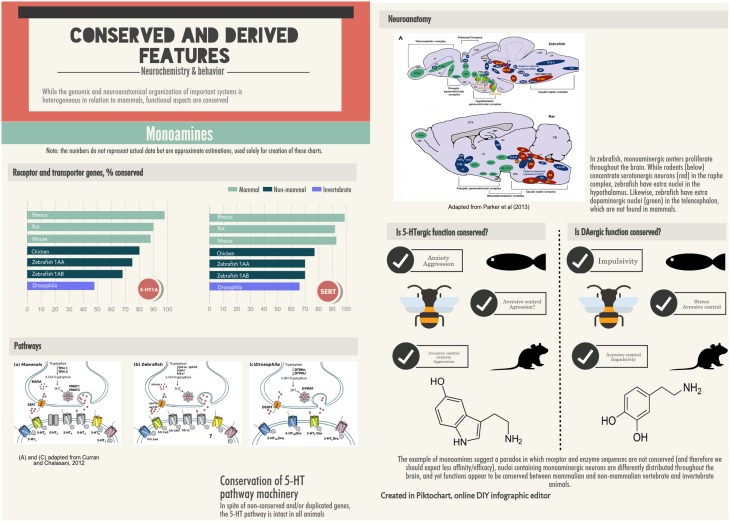
**Apparent paradox in the divergence and conservation of monaminergic systems in mammals, fish, and insects**. Receptor and enzyme sequences are not conserved (including gene duplication in the case of teleost fish), and the brain nuclei containing monoaminergic neurons are differently distributed throughout the brain in mammals, fish, and insects, but functions appear to be relatively well-conserved.

The exclusive reliance of behavioral neurosciences on a small number of species is counterproductive; as a result, different research groups are starting to focus their efforts on a comparative perspective, using non-mammalian organisms in research. As can be inferred from this Review, the most readily transferable models using non-mammalian species are in the domains of anxiety, impulse control and aggression. While our knowledge of the neurobehavioral systems involved in disorders in these domains is far from complete, mammalian data suggests that a number of neurotransmitter and neuromodulator systems are dysfunctional in these pathologies (Figure 3). In Tables 1–3 and throughout this Review, the degree of conservation in these systems has been discussed. In some cases, a seemingly paradoxical situation is seen, in which the neurotransmitter system is not fully conserved from the molecular point of view, but from a functional perspective the degree of conservation is higher (Figure 3). While this state of affairs can simply represent the current limitations in the tools used to address these questions, it is possible that they represent discontinuities in the evolutionary histories of these traits. Future research—especially of the comparative kind—will answer these open questions.

Each organism brings advantages and disadvantages, some of which we have discussed in the present Review. As is the case with rodents, no single species can be used to answer all research questions, and there is no such thing as a “perfect laboratory organism.” Ideally, all behavioral research using these species should be inserted into a comparative framework, assessing the same variables on different species and/or trying to extrapolate findings in different species; nonetheless, while at the present moment most research using non-mammalian organisms relies on data produced in rodents, the inverse is not necessarily true. The widespread adoption of other species is advantageous from a comparative and epistemic point of view, but still needs to go a long way to impact the field.

### Conflict of interest statement

The authors declare that the research was conducted in the absence of any commercial or financial relationships that could be construed as a potential conflict of interest.
